# Association of chemerin with oxidative stress, inflammation and classical adipokines in non-diabetic obese patients

**DOI:** 10.1111/jcmm.12282

**Published:** 2014-04-06

**Authors:** Péter Fülöp, Ildikó Seres, Hajnalka Lőrincz, Mariann Harangi, Sándor Somodi, György Paragh

**Affiliations:** Division of Metabolic Diseases, Department of Internal Medicine, University of Debrecen Medical and Health Science CenterDebrecen, Hungary

**Keywords:** obesity, chemerin, leptin, adiponectin, oxidized low-density lipoprotein, paraoxonase-1, inflammation

## Abstract

The prevalence of obesity has been increasing worldwide. Chemerin is a recently discovered adipokine secreted by the enlarged adipose tissue with diverse biological effects that are not well detailed yet. This study aimed to elucidate the potential role of chemerin in oxidative stress and inflammation that are characteristics for excess weight and may eventually lead to insulin resistance and atherosclerotic complications. We also analysed the associations between chemerin and classical adipokines, namely leptin and adiponectin. Therefore, we investigated non-diabetic obese patients without manifest cardiovascular disease and compared their data to healthy lean individuals. Chemerin correlated positively with markers of oxidative stress and inflammation, while it showed a negative correlation with the measure of antioxidant status, characterized by the HDL-linked paraoxonase-1 enzyme. Chemerin also correlated positively with leptin and negatively with adiponectin respectively. In our study population, oxidized low-density lipoprotein and high-sensitivity C-reactive protein were found to be the strongest predictors of chemerin level. We conclude that chemerin may contribute to chronic inflammation and increased oxidative stress in obese individuals, even in the absence of manifest insulin resistance.

## Introduction

Obesity and overweight represent major health burdens worldwide. Excess weight is an established cardiovascular risk factor and body mass index (BMI) correlates positively with cardiovascular mortality [1]. White adipose tissue, especially in the visceral compartment, is recently considered as not a simple energy depository tissue, but also as an active endocrine organ releasing a variety of biologically active substances termed adipokines. Generally, adipokines are known to play key roles in the regulation of glucose/lipid metabolism, insulin sensitivity and inflammation. Based upon the complex interplay between adipokines, obesity is also characterized by a chronic low-grade inflammation with permanently increased oxidative stress [2]. The imbalance between oxidative stress and antioxidant defence also triggers insulin resistance and results in enhanced atherosclerosis [3]. Oxidative stress is also a hallmark of obesity-related dyslipidaemia leading to cardiovascular diseases by decreasing aortic flow and left ventricular function, while increasing the extent of myocardial necrosis under experimental settings [4].

Chemerin, named also as tazarotene-induced gene protein 2 or retinoic acid receptor responder protein 2 (RARRES2), is a novel adipokine with biological functions that are still not elucidated yet. The protein and its receptor (chemokine-like receptor 1, CMKLR1 or ChemR23) are highly expressed in the adipose tissue [5], and chemerin is reported to regulate adipocyte differentiation and metabolism in an autocrine/paracrine manner [6]. Chemerin also serves as a chemoattractant for immune cells such as macrophages, natural killer cells and dendritic cells [7]. Its exact effect on inflammation is still not clear, as it might serve as a pro- and anti-inflammatory protein too [8]. Recent data show that chemerin might play a role in the development of obesity and metabolic syndrome [5,9]. Chemerin may also impair glucose uptake and promote insulin resistance [10]; and its level was reported to be associated positively with BMI and the markers of inflammation and metabolic syndrome in humans [11,12]. Genetic studies also indicated that chemerin might be involved in adipose tissue homoeostasis with increased lipogenic activity, reporting that aged ChemR23 knockout mice were prone to develop mild obesity without major defects in adipocyte differentiation [13]. The above mentioned conditions are also characterized by increased oxidative stress; however, the role of chemerin in such oxidative processes still remains unknown.

The association between chemerin and classical adipokines, such as leptin and adiponectin has also yet to be elucidated. Leptin was reported to induce pro-inflammatory responses and its serum levels were shown to increase with the amount of body fat [14,15]. In fact, leptin was found to contribute to the development of cardiac failure in rats with experimental myocardial infarction, possibly by increasing intramyocardial pro-inflammatory cytokine expression [16]. In contrast, adiponectin levels are decreased in obesity and correlate inversely with the risk of myocardial infarction [17]. In addition, adiponectin promotes insulin sensitivity and the production of anti-inflammatory cytokines [18].

Altered lipoprotein profile, generally termed as atherogenic dyslipidaemia, is often found in obese individuals. Oxidation of low-density lipoprotein (LDL) plays a key role in atherosclerosis [19], eventually leading to the development of various cardiovascular diseases. Oxidized LDL (ox-LDL) initiates monocyte/macrophage and endothelial activation, smooth muscle cell proliferation and formation of foam cells and fatty streaks [20]. Besides triggering inflammation, ox-LDL is considered to be a useful marker for cardiovascular diseases, as literature data indicate that plasma ox-LDL level is significantly elevated in such patients [3,21]. Indeed, levels of ox-LDL were found to correlate positively with the severity of acute coronary syndrome and more severe atherosclerotic lesions contained a significantly increased number of ox-LDL-positive macrophages [22], indicating the importance of ox-LDL in the latter stages of the atherosclerotic process too.

Activation of the natural antioxidant defence mechanisms may slow down the progression of atherosclerosis. In fact, enhancing antioxidant capacity exhibited cardioprotective effects and was shown to be effective in reducing atherosclerotic plaque formation in rodents [23,24]. Human paraoxonases are a group of enzymes encoded on chromosome 7 that mediate the hydrolysis of organophosphates. Paraoxonase (PON)1 is a HDL-linked enzyme with established antioxidant properties that protects LDL and HDL from oxidative modification [25–27]. PON2 is expressed ubiquitously and may also act as an antioxidant [28], while HDL-bound PON3 has recently been described to possess putative antioxidant effects [29]. PON1 is the most extensively described member of this gene family with several different enzyme activities, such as esterase, peroxidase and lactonase activities respectively [30]. Of note, PON1 arylesterase activity was reported to correlate inversely with the risk of major adverse cardiovascular events [31]. As it is reviewed elsewhere, PON1 status is reported to be reduced in several human diseases involving enhanced oxidative stress, such as diabetes mellitus, hyperlipidaemia, ischaemic heart disease and chronic liver failure [32,33]. Indeed, our previous data indicated that PON1 activity associated negatively with markers of metabolic syndrome [34]. We also reported that PON1 activities were decreased in obese children and PON1 arylesterase activity showed variable correlations with adipokine levels [35]. Variations of PON1 activities are mainly related to a common polymorphism (Q192R) in its coding region [36]. This Gln192Arg polymorphism yields three PON1 phenotypes with different enzymatic activities: AA (low activity), AB (intermediate activity) and BB (high activity) respectively. Q192R polymorphism was also shown to be associated with an increased risk for development of obesity in humans [37].

It should also be noted that a significant proportion of obese individuals are considered non-diabetic with normal insulin sensitivity. In a recent study, metabolically healthy obese individuals were reported to have lower risk of mortality and cardiovascular disease compared to metabolically unhealthy insulin resistant counterparts [38]; however, obese individuals still possess a significant risk of type 2 diabetes mellitus [39]. This suggests that mechanisms other than simple caloric imbalance, such as oxidative stress, antioxidant status, chronic inflammation and adipokine interplay might determine the clinical outcome in obese individuals.

To our knowledge, there is no data about the association between the levels of chemerin and ox-LDL, especially in non-diabetic obese (NDO) individuals. Literature about correlation of chemerin levels and paraoxonase-1 status is also lacking in these individuals. Therefore, we aimed to investigate the above mentioned variables in NDO patients and compared their data to non-diabetic lean individuals. We also intended to clarify the possible associations between chemerin levels and various markers of obesity, including lipid profile, high-sensitivity C-reactive protein (hsCRP) and classical adipokines such as leptin and adiponectin in these patients.

## Patients and methods

### Study population

The study was carried out in accordance with the Declaration of Helsinki of World Medical Association and was previously approved by the local and regional ethics committees. All investigated patients gave their written informed consent to participate in the study. We enrolled 50 consecutive NDO patients that were referred to our obesity outpatient clinic at Department of Internal Medicine, University of Debrecen Medical and Health Center, Debrecen, Hungary; and compared their data to 38 non-diabetic non-obese control participants matched in age and gender, that were recruited from our department. Patients with active liver or endocrine disease (including any type of diabetes mellitus), cardiovascular disease, renal impairment, malignancy, alcohol or drug dependence were excluded. The study population was also limited to non-smoker, non-pregnant individuals free of clinically significant infectious diseases. Neither obese patients nor non-obese controls were taking lipid lowering, hypoglycaemic, anti-inflammatory, antithrombotic medications or dietary supplements. None of the studied individuals were receiving antihypertensive treatment with the exception of 10 NDO patients, who were on diuretics (indapamide) because of mild hypertension. BMI was calculated as a ratio of the weight to the square of the height in SI measurements (kg/m^2^) and obesity was defined as BMI ≥ 30 kg/m^2^.

### Biochemical assays

Venous blood samples were taken after an overnight fast and sera were prepared immediately. Routine laboratory analyses [hsCRP, fructosamine, triglyceride, total cholesterol, HDL-cholesterol (HDL-C), LDL-cholesterol (LDL-C), apolipoprotein A1 (Apo A1), apolipoprotein B (Apo B), lipoprotein (a) [Lp(a)], haemoglobin A1C (HbA1C) and insulin levels] were performed from fresh sera with Cobas c501 autoanalyzer (Roche Ltd., Mannheim, Germany) in the Department of Laboratory Medicine of our university. Reagents were purchased from the same vendor and the tests were performed according to the recommendation of the manufacturer. To confirm non-diabetic status in the studied individuals, we applied a routine 75-g oral glucose tolerance test (OGTT) that was performed after an overnight fast, using capillary glucose measurements in our laboratory. Homoeostasis model assessment for insulin resistance (HOMA-IR) was calculated as described elsewhere [40].

### Ox-LDL assay

Serum concentrations of ox-LDL were detected by a commercially available solid phase two-site enzyme immunoassay kit (Mercodia AB, Uppsala, Sweden). Measurements of the oxidized LDL levels in the sera were performed according to the recommendations of the manufacturer. The intra- and inter-assay coefficients of variations were 5.5–7.3% and 4.0–6.2%, respectively, and the sensitivity was <1 mU/l.

### Paraoxonase-1 measurements

Paraoxonase-1 paraoxonase activity was analysed by a kinetic, semi-automated method. Briefly, we used paraoxon (O,O-diethyl-O-p-nitrophenyl-phosphate, Sigma, Hungary) as a substrate, and the generation of 4-nitrophenol was measured on a microtiter plate (Greiner Bio-One GmbH, Frickenhausen, Germany). Serum of 15 μl was mixed with 285 μl Tris-HCl buffer (100 mmol/l, pH = 8.0) containing 2 mmol/l CaCl_2_ and 5.5 mmol/l paraoxon. The absorbance was monitored at 405 nm (25°C), in every minute for 6 min. by a Beckman Coulter DTX880 Plate Reader (Beckman Coulter, Brea, CA, USA) equipped with a multimode detector. Enzyme activity was calculated using the molar extinction coefficient 17,600 M/cm. Paraoxonase activity is expressed as units per litre of serum, where 1 unit equals 1 μmol of substrate hydrolysed per minute.

Paraoxonase-1 arylesterase activity was assayed containing 1 mM phenylacetate substrate (Sigma-Aldrich, Budapest, Hungary) in 20 mM Tris-HCl, pH = 8.0. The reaction was started by adding the serum and the absorbance was monitored at 270 nm. Blanks were included to correct for the spontaneous hydrolysis of phenylacetate. Enzyme activity was calculated using the molar extinction coefficient 1310 M/cm. Arylesterase activity is expressed in U/l; 1 U is defined as 1 μmol phenylacetate hydrolysed per minute.

### Adipokine measurements

Serum concentrations of leptin, adiponectin and chemerin were measured by a commercially available ELISA kits (R&D Systems Europe Ltd., Abington, UK for leptin and adiponectin determinations; USCN Life Science Inc., Wuhan, China, for chemerin measurements). The intra- and inter-assay coefficients of variations were 3.0–3.3% and 3.5–5.4% (leptin), 2.5–4.7% and 5.8–6.9% (adiponectin), <10% and <12% (chemerin) respectively. Measurements of the adipokine levels in sera were performed according to the recommendations of the manufacturers.

### Statistical analysis

Statistical analysis was performed by SAS™ for Windows™ 8.2 computer software (SAS Institute Inc., Cary, NC, USA). Normality of distribution was tested by Kolmogorov–Smirnov test. Data are expressed as means ± SD in parameters with normal distribution; and as median (lower/upper quartile) in case of non-normal distribution. Comparisons between groups (NDO patients *versus* healthy controls) were analysed by Student's unpaired *t*-tests in parameters showing normal distribution and Mann–Whitney *U*-tests were performed to compare parameters with non-normal distribution. Correlations between continuous variables were assessed by calculation of linear regression using Pearson's test. Multiple regression analysis (backward-stepwise method) was performed to determine variables best predicted chemerin levels. *P* < 0.05 was considered statistically significant.

## Results

The characteristics of the study participants are shown at Table 1. As expected, classical adipokines such as leptin and adiponectin showed opposite changes in their levels: leptin was significantly increased in NDO patients, while adiponectin was measured to be significantly decreased in these patients compared to control individuals. In line with leptin, chemerin was also found to be elevated in NDO patients, together with significantly increased hsCRP levels. The other routinely measured metabolic parameters were in the physiological range, however, triglyceride, HDL-C, Apo A1 and Lp(a) concentrations were significantly altered in the NDO group. In our study patients, normal insulin sensitivity was confirmed by several assays, as OGTT, HbA1C, fasting insulin levels and HOMA-IR were normal in both groups. Ox-LDL level was significantly increased in the NDO individuals, while PON1 paraoxonase and arylesterase activities did not show significant differences across the groups.

**Table 1:** Anthropometric and selected laboratory parameters of the study population

Variables	NDO (*n* = 50)	Control (*n* = 38)	*P*
Gender (female/male)	43/7	33/5	
Age (years)	44.2 ± 13.5	42.3 ± 11.4	n.s.
BMI (kg/m^2^)	41.96 ± 8.63	24.05 ± 3.21	<0.001
Leptin (ng/ml)	69.09 ± 44.19	15.87 ± 12.11	<0.001
Adiponectin (μg/ml)	6.24 ± 3.30	10.11 ± 5.94	<0.001
Chemerin (ng/ml)	590.08 ± 90.29	404.99 ± 127.07	<0.001
hsCRP (mg/l)	8.24 (3.2/13.09)	0.85 (0.5/1.38)	<0.001
Fructosamine (μmol/l)	225.32 ± 27.95	233.90 ± 15.76	n.s.
Triglyceride (mmol/l)	1.4 (1.1/2.0)	0.9 (0.7/1.6)	<0.01
Total cholesterol (mmol/l)	5.04 ± 0.83	5.06 ± 1.03	n.s.
HDL-C (mmol/l)	1.36 ± 0.33	1.72 ± 0.47	<0.001
LDL-C (mmol/l)	3.17 ± 0.74	2.93 ± 0.98	n.s.
Apo A1 (g/l)	1.48 ± 0.24	1.66 ± 0.39	<0.01
Apo B (g/l)	0.86 ± 0.20	0.78 ± 0.26	n.s
Lp(a) (mg/l)	248 (120/586)	112 (80/300)	<0.01
Glucose – OGTT 0 min.	4.90 ± 0.75	4.41 ± 0.57	<0.01
Glucose – OGTT 120 min.	7.00 ± 2.01	6.54 ± 1.13	n.s.
HbA1C (%)	5.81 ± 0.57	5.90 ± 0.63	n.s.
Insulin (mU/l)	17.14 ± 9.91	14.26 ± 6.02	n.s.
HOMA-IR	3.2 (2.2/5.1)	2.4 (1.3/3.3)	n.s.
Ox-LDL (U/l)	46.8 ± 9.94	39.7 ± 10.40	<0.001
PON1 paraoxonase activity (U/l)	82.0 (45.1/149.5)	79.7 (40.0/200.0)	n.s.
PON1 arylesterase activity (U/l)	113.95 ± 24.90	129.19 ± 27.01	n.s.

Data are presented as mean ± SD or as median (lower/upper quartile). p: NDO patients *versus* control individuals.

Ox-LDL demonstrated a significant positive correlation with chemerin (*r* = 0.401, *P* = 0.001), and a significant negative correlation with adiponectin (*r* = −0.366, *P* = 0.004). The correlation was non-significant between ox-LDL and leptin (*r* = −0.138, *P* = 0.346). We also detected significant positive correlations between concentrations of chemerin and LDL-C (*r* = 0.284, *P* = 0.008), and between concentrations of chemerin and Apo B (*r* = 0.349, *P* = 0.001) respectively; while significant negative correlations were found between levels of chemerin and HDL-C (*r* = −0.296, *P* = 0.0057), and between levels of chemerin and Apo A1 (*r* = −0.237, *P* = 0.0298) respectively.

We also evaluated the PON1 phenotype distribution and the allelic frequencies that are depicted on Table 2. Allelic frequencies followed the Hardy–Weinberg equilibrium and no significant differences were found between the studied groups.

**Table 2:** Paraoxonase-1 phenotype distribution and allelic frequencies

	NDO (*n* = 50)	Control (*n* = 38)
	*n* (%)	*n* (%)
AA	45 (90)	31 (82)
AB	5 (10)	7 (18)
BB	0 (0)	0 (0)
A	95 (95)	69 (91)
B	5 (5)	7 (9)

AA, AB, BB: paraoxonase-1 phenotype distribution representing different enzymatic activities (AA phenotype: low activity; AB phenotype: intermediate activity; BB phenotype: high activity). A: allele A; B: allele B.

**Fig. 1:**
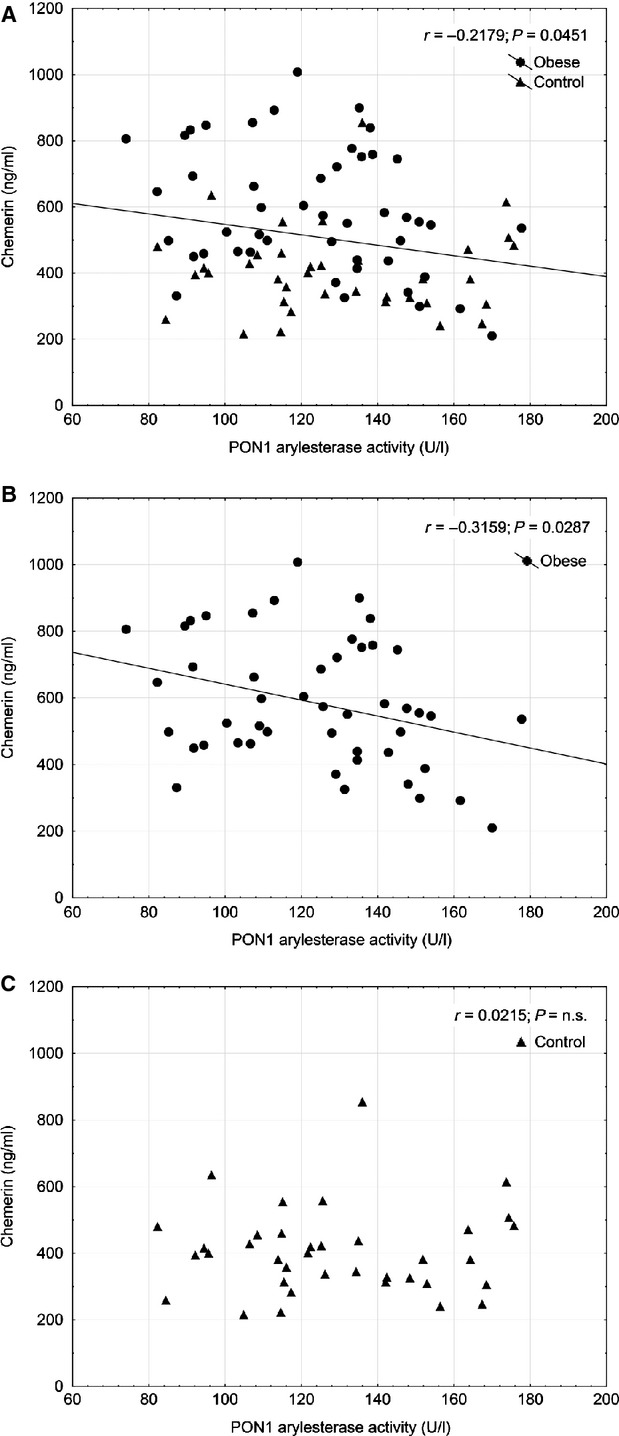
Association of PON1 arylesterase activity with chemerin in all studied individuals (**A**), in non-diabetic obese patients (**B**) and in lean controls (**C**).

Although PON1 paraoxonase activity was not found to be associated with chemerin level (*r* = −0.133, *P* = 0.357), we detected a significant negative correlation between PON1 arylesterase activity and chemerin concentration (*r* = −0.218, *P* = 0.045) as it is shown on Figure 1. The arylesterase activity of PON1 characterizes the antioxidant capacity and the quantity of the enzyme bound to HDL. The negative correlation found in the whole study population persisted and remained significant when analysing the obese patients only (*r* = −0.316, *P* = 0.029) and we found PON1 arylesterase activity to be unrelated to chemerin level in healthy controls (*r* = 0.022, *P* = 0.899).

**Fig. 2:**
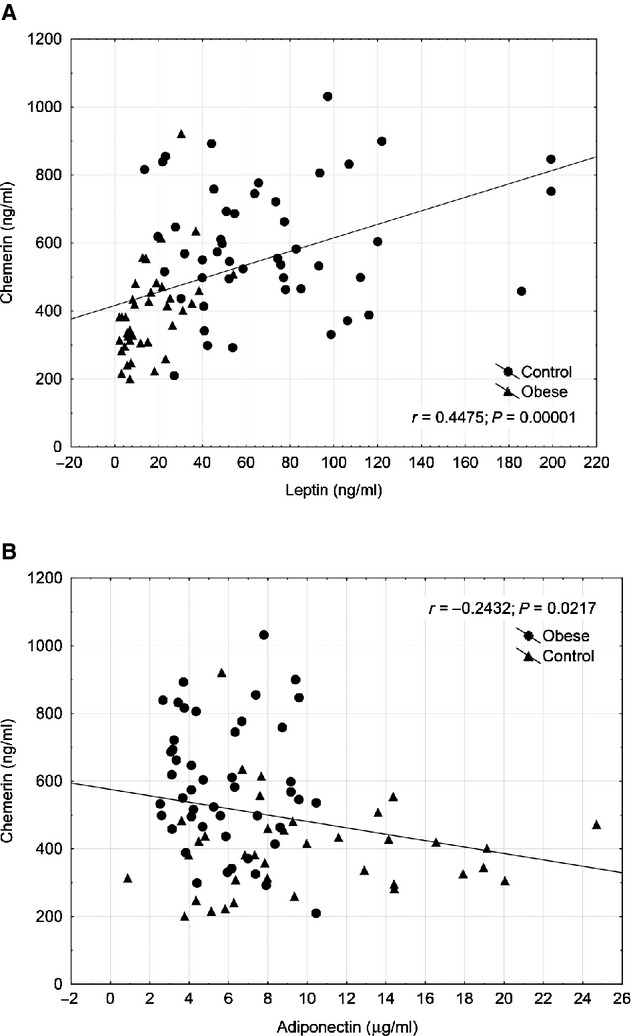
Association of chemerin with leptin (**A**) and adiponectin (**B**).

Body mass index correlated positively with leptin (*r* = 0.760, *P* < 0.0001) and negatively with adiponectin levels (*r* = −0.401, *P* = 0.0001). Body mass index also showed a significant positive correlation with chemerin levels (*r* = 0.546, *P* < 0.0001). We also found a significant positive correlation between the levels of hsCRP and chemerin (*r* = 0.610, *P* < 0.0001). As previous data were confounding, we also determined the associations between chemerin and formerly discovered adipokines. Chemerin might either act as a pro- or anti-inflammatory molecule, while leptin and adiponectin have opposing effect on inflammation and cardiac health. Assessing the correlations between chemerin, as a novel adipokine and the formerly discovered classical adipokines, we found a significant positive correlation between concentrations of leptin and chemerin (*r* = 0.448, *P* = 0.00001) as it is demonstrated on Figure 2. Also, a significant negative correlation was detected between adiponectin and chemerin levels (*r* = −0.243, *P* = 0.022).

To test whether the associations detected in the univariate analyses were independent of anthropometric and other laboratory parameters, we carried out multiple regression analysis with chemerin as the dependent variable. The model included age, BMI, hsCRP, HDL-C, leptin, adiponectin, PON arylesterase and ox-LDL. As it is shown on Table 3, chemerin turned out to be best predicted by ox-LDL and hsCRP. There were also tendencies for significance in correlations with age and PON1 arylesterase activity; however, these associations did not reach the level of significance.

**Table 3:** Multiple regression analysis for chemerin as a dependent variable

Variable	β	*P*
Ox-LDL	0.381	0.005
hsCRP	0.284	0.029
Age	0.230	0.068
PON1 arylesterase activity	−0.230	0.075
BMI	0.175	0.302
HDL-C	−0.136	0.411
Adiponectin	0.121	0.438
Leptin	0.114	0.475

## Discussion

Recent evidence demonstrates the role of chemerin in the development of obesity-related insulin resistance, while dyslipidaemia and inflammation are known to enhance atherosclerosis [10,41]; however, data are conflicting. In our study, despite unaltered insulin sensitivity, we found significantly increased chemerin and leptin levels and significantly decreased adiponectin concentrations in obese patients. Although lipid parameters were in the normal range in our study, the adipokine pattern of NDO patients was paralleled by atherogenic dyslipidaemia, compared to lean patients. Significantly increased hsCRP levels confirmed the chronic low-grade inflammation in NDO patients that is also characteristic for obesity [42]. Our results indicate the early presence of unfavourable pro-atherogenic processes in NDO patients that might eventually lead to cardiovascular disease or insulin resistance.

The adipokine profile of our patients is in agreement with previous data suggesting that circulating chemerin level is increased in obesity and might play a key role in obesity-related disorders [8]. Indeed, chemerin concentration was found to be increased in nascent metabolic syndrome [9] and it also showed significant correlations with altered lipid parameters [43]. We also detected significant positive correlations between the concentrations of pro-atherogenic lipid parameters (LDL-C, Apo B) and chemerin; while significant negative correlations were found between the levels of anti-atherogenic lipid measures (HDL-C, Apo A1) and chemerin respectively. Our findings also imply that chemerin might play an important role in atherosclerosis.

In congruence with dyslipidaemia, ox-LDL was significantly elevated in the NDO individuals. In turn, PON1 paraoxonase and arylesterase activities did not show significant differences across the groups, indicating the early imbalance between pro-oxidant mechanisms and antioxidant defence. Although obesity and dyslipidaemia are characterized by increased oxidative stress, data are extremely scarce and confounding about the *in vivo* associations of chemerin and oxidative status. Ox-LDL is a key lipid peroxidation product generated in the early stages of atherosclerosis [19]. Indeed, we found increased oxidized LDL levels in NDO patients, which demonstrated a significant positive correlation with chemerin and a significant negative correlation with adiponectin. These novel data support the assumption that chemerin might be involved in increased oxidative stress that is observed often in these patients.

As dyslipidaemia, increased oxidative stress and unfavourable adipokine profile was found in the NDO patients together with absence of altered paraoxonase activities, we evaluated the PON1 phenotype distribution and the allelic frequencies. Our findings were in accordance with the results of our and other research groups [44,45]. No significant differences were found between the studied groups indicating the same prevalence of PON1 Q192R polymorphism in our patients. PON1 arylesterase activity correlated negatively with chemerin concentration when analysing the whole study population or the NDO individuals, which indicates the impact of obesity on antioxidant capacity even in the absence of manifest insulin resistance or cardiovascular complications. To the best our knowledge, this is the first report about the inverse relationship between chemerin and the HDL-linked paraoxonase. This, together with our novel results regarding chemerin and its association with ox-LDL, may support the impact of chemerin on oxidant/antioxidant status in obesity. PON1 arylesterase activity is responsible for the hydrolysis of phospholipid and cholesterol ester hydroperoxides and it is proportional to the quantity of PON1 enzyme protein linked to HDL [46]. Ox-LDL is also known to inactivate PON1 arylesterase [47]. Despite the increased ox-LDL levels observed in the obese patients, we found slightly, but not significantly decreased arylesterase activity in these individuals. Taken into account that PON1 is produced by the liver and chemerin also shows a high expression in this organ of rats and humans especially in non-alcoholic steatosis that is closely associated with obesity [5,48], it is tempting to speculate that chemerin might modify the synthesis of PON1 in the hepatocytes of the obese patients. However, further studies are needed to clarify this putative mechanism.

Corroborating previous data [49], BMI correlated positively with leptin levels and negatively with adiponectin concentrations. Body mass index also showed a significant positive correlation with chemerin levels. Chemerin mRNA is abundantly expressed in white adipose tissue in humans and its receptor is mainly expressed in immune and fat cells [5], raising the link between obesity and inflammation. Chemerin is secreted as an 18 kDa inactive form that undergoes further enzymatic proteolysis generating either pro-inflammatory and chemotactic effects or anti-inflammatory signalling [50]. In our study patients, we found a significant positive correlation between the levels of hsCRP and chemerin, indicating the possible modulating role of chemerin in inflammation.

Assessing the correlations between chemerin, as a novel adipokine and the formerly discovered classical adipokines, we found a significant positive correlation between concentrations of leptin and chemerin. Also, a significant negative correlation was detected between adiponectin and chemerin levels. Although previous studies found no association between chemerin and adiponectin in patients with various BMI [12,51], here we report a negative correlation of chemerin to adiponectin, which, together with the positive association with leptin, may further support the potential role of chemerin in the development of obesity-related disorders. As adiponectin and leptin are considered to have opposing effects on inflammation in obesity [52], these findings rather indicate a pro-inflammatory role of chemerin in NDO patients.

Based upon our multiple regression analysis, chemerin turned out to be best predicted by ox-LDL and hsCRP. These novel findings also highlight the importance of chemerin in inflammation and reveal its possible role in the regulation of oxidant/antioxidant balance. Our data indicate that obesity, even without manifest insulin resistance, predisposes to enhanced atherosclerosis and increased oxidative stress, in which chemerin functions as a potential modulator of inflammation and oxidant/antioxidant status. Our results support the hypothesis that circulating chemerin levels are already elevated in NDO patients that are free of manifest insulin resistance and cardiovascular diseases. The early presence of low-grade inflammation and oxidative stress modulated by chemerin predisposes to accelerated atherogenesis in obesity. Therefore, chemerin might serve as an early biomarker for increased cardiovascular risk in obese patients without manifest complications and might be a useful tool for timely intervention to prevent the development of subsequent atherosclerotic complications. However, further studies are needed to clarify the role of chemerin in the long-term cardiovascular outcome in obese patients.

## References

[1] Calle EE, Thun MJ, Petrelli JM (1999). Body-mass index and mortality in a prospective cohort of U.S. adults. N Engl J Med.

[2] Ntaios G, Gatselis NK, Makaritsis K (2013). Adipokines as mediators of endothelial function and atherosclerosis. Atherosclerosis.

[3] Hulsmans M, Holvoet P (2010). The vicious circle between oxidative stress and inflammation in atherosclerosis. J Cell Mol Med.

[4] Kertész A, Bombicz M, Priksz D (2013). Adverse impact of diet-induced hypercholesterolemia on cardiovascular tissue homeostasis in a rabbit model: time-dependent changes in cardiac parameters. Int J Mol Sci.

[5] Bozaoglu K, Bolton K, McMillan J (2007). Chemerin is a novel adipokine associated with obesity and metabolic syndrome. Endocrinology.

[6] Goralski KB, McCarthy TC, Hanniman EA (2007). Chemerin, a novel adipokine that regulates adipogenesis and adipocyte metabolism. J Biol Chem.

[7] Wittamer V, Franssen JD, Vulcano M (2003). Specific recruitment of antigen-presenting cells by chemerin, a novel processed ligand from human inflammatory fluids. J Exp Med.

[8] Roman AA, Parlee SD, Sinal CJ (2012). Chemerin: a potential endocrine link between obesity and type 2 diabetes. Endocrine.

[9] Jialal I, Devaraj S, Kaur H (2013). Increased chemerin and decreased omentin-1 in both adipose tissue and plasma in nascent metabolic syndrome. J Clin Endocrinol Metab.

[10] Sell H, Laurencikiene J, Taube A (2009). Chemerin is a novel adipocyte-derived factor inducing insulin resistance in primary human skeletal muscle cells. Diabetes.

[11] Bozaoglu K, Segal D, Shields KA (2009). Chemerin is associated with metabolic syndrome phenotypes in a Mexican-American population. J Clin Endocrinol Metab.

[12] Lehrke M, Becker A, Greif M (2009). Chemerin is associated with markers of inflammation and components of the metabolic syndrome but does not predict coronary atherosclerosis. Eur J Endocrinol.

[13] Rouger L, Denis GR, Luangsay S (2013). ChemR23 knockout mice display mild obesity but no deficit in adipocyte differentiation. J Endocrinol.

[14] Kloek C, Haq AK, Dunn SL (2002). Regulation of Jak kinases by intracellular leptin receptor sequences. J Biol Chem.

[15] Considine RV, Sinha MK, Heiman ML (1996). Serum immunoreactive-leptin concentrations in normal-weight and obese humans. N Engl J Med.

[16] Moro C, Grauzam S, Ormezzano O (2011). Inhibition of cardiac leptin expression after infarction reduces subsequent dysfunction. J Cell Mol Med.

[17] Pischon T, Girman CJ, Hotamisligil GS (2004). Plasma adiponectin levels and risk of myocardial infarction in men. JAMA.

[18] Díez JJ, Iglesias P (2003). The role of the novel adipocyte-derived hormone adiponectin in human disease. Eur J Endocrinol.

[19] Steinberg D (1997). Low density lipoprotein oxidation and its pathobiological significance. J Biol Chem.

[20] Luc G, Fruchart JC (1991). Oxidation of lipoproteins and atherosclerosis. Am J Clin Nutr.

[21] Toshima S, Hasegawa A, Kurabayashi M (2000). Circulating oxidized low density lipoprotein levels. A biochemical risk marker for coronary heart disease. Arterioscler Thromb Vasc Biol.

[22] Ehara S, Ueda M, Naruko T (2001). Elevated levels of oxidized low density lipoprotein show a positive relationship with the severity of acute coronary syndromes. Circulation.

[23] Bak I, Lekli I, Juhasz B (2006). Cardioprotective mechanisms of Prunus cerasus (sour cherry) seed extract against ischemia-reperfusion-induced damage in isolated rat hearts. Am J Physiol Heart Circ Physiol.

[24] Juhasz B, Kertész A, Balla J (2013). Cardioprotective effects of sour cherry seed extract (SCSE) on the hypercholesterolemic rabbit heart. Curr Pharm Des.

[25] Getz GS, Reardon CA (2004). Paraoxonase, a cardioprotective enzyme: continuing issues. Curr Opin Lipidol.

[26] Watson AD, Berliner JA, Hama SY (1995). Protective effect of high density lipoprotein associated paraoxonase. Inhibition of the biological activity of minimally oxidized low density lipoprotein. J Clin Invest.

[27] Aviram M, Rosenblat M, Bisgaier CL (1998). Paraoxonase inhibits high-density lipoprotein oxidation and preserves its functions. A possible peroxidative role for paraoxonase. J Clin Invest.

[28] Ng CJ, Wadleigh DJ, Gangopadhyay A (2001). *et al*. Paraoxonase-2 is a ubiquitously expressed protein with antioxidant properties and is capable of preventing cell-mediated oxidative modification of low density lipoprotein. J Biol Chem.

[29] Kempster SL, Belteki G, Licence D (2012). Disruption of paraoxonase 3 impairs proliferation and antioxidant defenses in human A549 cells and causes embryonic lethality in mice. Am J Physiol Endocrinol Metab.

[30] Rosenblat M, Gaidukov L, Khersonsky O (2006). The catalytic histidine dyad of high density lipoprotein-associated serum paraoxonase-1 (PON1) is essential for PON1-mediated inhibition of low density lipoprotein oxidation and stimulation of macrophage cholesterol efflux. J Biol Chem.

[31] Tang WH, Hartiala J, Fan Y (2012). Clinical and genetic association of serum paraoxonase and arylesterase activities with cardiovascular risk. Arterioscler Thromb Vasc Biol.

[32] Costa LG, Giordano G, Furlong CE (2011). Pharmacological and dietary modulators of paraoxonase 1 (PON1) activity and expression: the hunt goes on. Biochem Pharmacol.

[33] Litvinov D, Mahini H, Garelnabi M (2012). Antioxidant and anti-inflammatory role of paraoxonase 1: implication in arteriosclerosis diseases. N Am J Med Sci.

[34] Bajnok L, Seres I, Varga Z (2008). Relationship of serum resistin level to traits of metabolic syndrome and serum paraoxonase 1 activity in a population with a broad range of body mass index. Exp Clin Endocrinol Diabetes.

[35] Koncsos P, Seres I, Harangi M (2010). Human paraoxonase-1 activity in childhood obesity and its relation to leptin and adiponectin levels. Pediatr Res.

[36] Mackness B, Mackness MI, Arrol S (1998). Effect of the human serum paraoxonase 55 and 192 genetic polymorphisms on the protection by high density lipoprotein against low density lipoprotein oxidative modification. FEBS Lett.

[37] Veiga L, Silva-Nunes J, Melão A (2011). Q192R polymorphism of the paraoxonase-1 gene as a risk factor for obesity in Portuguese women. Eur J Endocrinol.

[38] Ortega FB, Lee DC, Katzmarzyk PT (2013). The intriguing metabolically healthy but obese phenotype: cardiovascular prognosis and role of fitness. Eur Heart J.

[39] Soriguer F, Gutiérrez-Repiso C, Rubio-Martín E (2013). Metabolically healthy but obese, a matter of time? Findings from the prospective Pizarra study. J Clin Endocrinol Metab.

[40] Matthews DR, Hosker JP, Rudenski AS (1985). Homeostasis model assessment: insulin resistance and beta-cell function from fasting plasma glucose and insulin concentrations in man. Diabetologia.

[41] Manduteanu I, Simionescu M (2012). Inflammation in atherosclerosis: a cause or a result of vascular disorders?. J Cell Mol Med.

[42] Visser M, Bouter LM, McQuillan GM (1999). Elevated C-reactive protein levels in overweight and obese adults. JAMA.

[43] Fatima SS, Bozaoglu K, Rehman R (2013). Elevated chemerin levels in pakistani men: an interrelation with metabolic syndrome phenotypes. PLoS ONE.

[44] Sztanek F, Seres I, Harangi M (2012). Decreased paraoxonase 1 (PON1) lactonase activity in hemodialyzed and renal transplanted patients. A novel cardiovascular biomarker in end-stage renal disease. Nephrol Dial Transplant.

[45] Rainwater DL, Rutherford S, Dyer TD (2009). Determinants of variation in human serum paraoxonase activity. Heredity.

[46] Cabana VG, Reardon CA, Feng N (2003). Serum paraoxonase: effect of the apolipoprotein composition of HDL and the acute phase response. J Lipid Res.

[47] Aviram M, Rosenblat M, Billecke S (1999). Human serum paraoxonase (PON 1) is inactivated by oxidized low density lipoprotein and preserved by antioxidants. Free Radic Biol Med.

[48] Döcke S, Lock JF, Birkenfeld AL (2013). Elevated hepatic chemerin mRNA expression in human non-alcoholic fatty liver disease. Eur J Endocrinol.

[49] Matsubara M, Maruoka S, Katayose S (2002). Inverse relationship between plasma adiponectin and leptin concentrations in normal-weight and obese women. Eur J Endocrinol.

[50] Yoshimura T, Oppenheim JJ (2008). Chemerin reveals its chimeric nature. J Exp Med.

[51] Weigert J, Neumeier M, Wanninger J (2010). Systemic chemerin is related to inflammation rather than obesity in type 2 diabetes. Clin Endocrinol.

[52] Kwon H, Pessin JE (2013). Adipokines mediate inflammation and insulin resistance. Front Endocrinol.

